# Non‐selective microbiota reduction after the elicitation of a seaweed's immune response

**DOI:** 10.1111/1758-2229.13268

**Published:** 2024-05-17

**Authors:** Jiasui Li, Mahasweta Saha, Marwan E. Majzoub, Teng Yang, Haiyan Chu, Torsten Thomas, Florian Weinberger, Suhelen Egan

**Affiliations:** ^1^ State Key Laboratory of Soil and Sustainable Agriculture, Institute of Soil Science Chinese Academy of Sciences Nanjing China; ^2^ Centre for Marine Science and Innovation, Faculty of Science, School of Biological, Earth and Environmental Sciences The University of New South Wales Kensington New South Wales Australia; ^3^ Marine Ecology Division GEOMAR Helmholtz Centre for Ocean Research Kiel Kiel Germany; ^4^ Marine Ecology and Biodiversity Plymouth Marine Laboratory Plymouth UK; ^5^ Faculty of Medicine and Health, School of Biomedical Sciences The University of New South Wales Kensington New South Wales Australia; ^6^ Institute of Soil Science University of Chinese Academy of Sciences Beijing China

## Abstract

Pattern‐triggered immunity (PTI) is an integral part of the innate immune system of many eukaryotic hosts, assisting in the defence against pathogen invasions. In plants and animals, PTI exerts a selective pressure on the microbiota that can alter community composition. However, the effect of PTI on the microbiota for non‐model hosts, including seaweeds, remains unknown. Using quantitative polymerase chain reaction complemented with 16S rRNA gene and transcript amplicon sequencing, this study profiled the impact that PTI of the red seaweed *Gracilaria gracilis* has on its microbiota. PTI elicitation with agar oligosaccharides resulted in a significant reduction in the number of bacteria (by >75% within 72 h after treatment). However, the PTI elicitation did not cause any significant difference in the community diversity or structure. These findings demonstrated that PTI can be non‐selective, and this might help to maintain a stable microbiota by uniformly reducing bacterial loads.

## Graphical abstract

We studied how the pattern‐triggered immunity (PTI) of a seaweed affects its epimicrobiota. We found that the seaweed's immunity reduced epimicrobiota abundance without altering the community diversity or structure. This suggests that the seaweed's innate immunity helps maintain a stable microbiota by regulating bacterial growth.

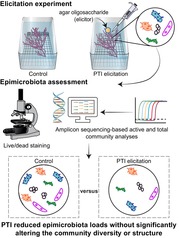

## INTRODUCTION

Macroorganisms are hosts to a wide variety of microorganisms that confer important functions and play a central role in their biology, ecology and evolution (Simon et al., 2019). A properly assembled microbiota (also referred to as ‘eubiotic’ microbiota; Paasch & He, 2021) is thus essential for host health and survival. In contrast, a disturbance to this balance, that is, a dysbiosis, can result in an increase in detrimental and/or a decrease in beneficial symbionts, thus leading compromised host health (Egan & Gardiner, 2016; Paasch & He, 2021).

Multiple mechanisms can be involved in maintaining microbiota eubiosis, including chemical signalling (Kessler et al., 2018; Lachnit et al., 2013; Lebeis et al., 2015) and inter‐species interaction (Li et al., 2022). For example, *Arabidopsis thaliana* produces the phytohormone salicylic acid, which can influence root colonization by certain bacterial families (Lebeis et al., 2015). Likewise, the green seaweed *Ulva mutabilis* produces dimethylsulfoniopropionate, which attracts bacteria that facilitate host development and morphogenesis (Kessler et al., 2018), while the kelp *Fucus vesiculosus* produces fucoxanthin, an antimicrobial compound that regulates the settlement of epiphytic bacteria (Lachnit et al., 2013).

In addition to such constitutive production of bioactive compounds, animals (Stuart et al., 2013), terrestrial (Hacquard et al., 2017) and aquatic vascular plants (Loucks et al., 2013), and some seaweeds (e.g., *Gracilaria* spp.) (Weinberger, 2007), have a pattern‐triggered immunity (PTI) that responds to two classes of chemical signals resulting from microbe–host interactions. The first class are the microbe‐associated molecular patterns (MAMPs), such as bacterial flagellin or fungal chitin, and the second are danger‐associated molecular patterns (DAMPs), which are altered‐self molecules resulting from the disruption of physical barriers, such as plant and seaweed cell walls by microbial invaders (Hacquard et al., 2017; Weinberger, 2007).

Activation of PTI results in a transient, yet rapid (within minutes) production of reactive oxygen species that is followed by regulatory phosphorylation events that lead to the expression of stress‐related genes, synthesis of defensive metabolites and the reinforcement of cell walls, over hours to days (Hacquard et al., 2017; Loucks et al., 2013; Stuart et al., 2013; Weinberger, 2007). Defects in parts of the PTI‐signalling pathway in *A. thaliana* and humans normally result in increased bacterial loads, lower diversity and altered species compositions, demonstrating a strongly selective role of PTI on the host microbiota (Chen et al., 2020; Lauro et al., 2016; Ma et al., 2021; Pfeilmeier et al., 2021). Elicitation of the PTI in seaweeds can also reduce microbial loads (e.g., Weinberger & Friedlander, 2000); however, to what extent this impacts microbial diversity or composition remains unknown. Compared with terrestrial plants, aquatic hosts are constantly exposed to a larger number and diversity of free‐living microorganisms (Sunagawa et al., 2015), thus they face different colonization pressures which could influence how PTI affects the host microbiota.

## EXPERIMENTAL PROCEDURES

**FIGURE 1:**
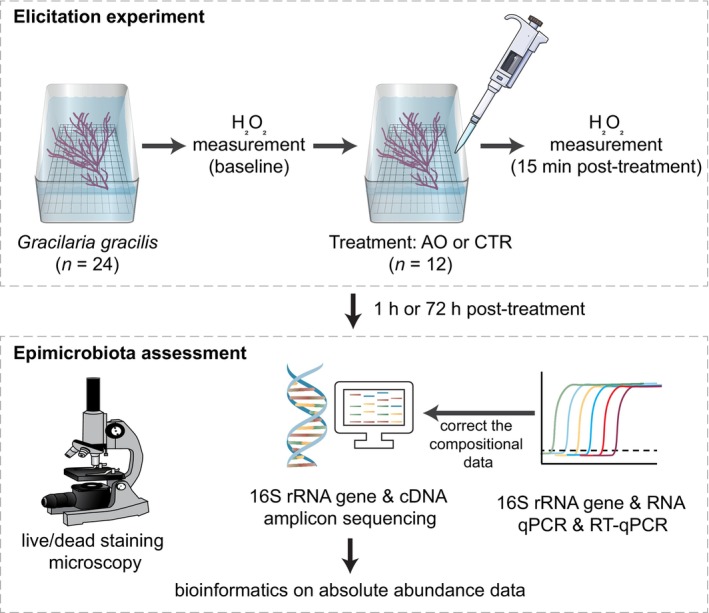
Experimental design. *Gracilaria gracilis* individuals are treated with either the danger‐associated molecular pattern agar oligosaccharide (AO) or sterile deionized water as a procedural control (CTR) and sampled after 1 h or 72 h for epimicrobiota assessment with different measures. Icons of this figure and graphical abstract are adapted from the Integration and Application Network Media Library (ian.umces.edu/media‐library, under a CC BY‐SA 4.0 license: https://creativecommons.org/licenses/by‐sa/4.0/), authored by Dieter Tracey (Department of Water Western Australia), Tracey Saxby, Jane Thomas, and Jane Hawkey (Integration and Application Network), and images from Servier Medical Art (https://smart.servier.com/, under a CC BY 3.0 license: https://creativecommons.org/licenses/by/3.0/).

To explore the influence of PTI on seaweed epimicrobiota, we performed a set of manipulative experiments to simulate DAMP‐triggered immunity elicitation in red seaweed *Gracilaria gracilis* and assess the changes in abundance, diversity and composition of its epimicrobiota (see Figure 1). Briefly, *G. gracilis* was treated with agar oligosaccharide (AO), which is a DAMP derived from the degradation of macroalgal cell walls (Weinberger & Friedlander, 2000), or sterile deionized water as a control (CTR). The seaweed epimicrobiota was collected after 1 h or 72 h by vortexing with glass beads in a sterile centrifuge tube with 50 mL of 0.22‐μm filtered seawater and vacuum filtering of 5 mL of the liquid onto 0.2‐μm polycarbonate filters. The filters were stained with the LIVE/DEAD™ BacLight™ Bacterial Viability Kit and examined by epifluorescence microscopy for a semi‐quantitative estimation of epibacteria. Total DNA and RNA were extracted from non‐vortexed tissue samples and used for 16S rRNA gene and transcript amplicon sequencing. The sequencing was performed on the Illumina MiSeq platform. The analysis of sequencing data was carried out with the USEARCH pipeline and analysed following the descriptions in (Li et al., 2022) and the Supporting information.

## RESULTS AND DISCUSSION

**FIGURE 2:**
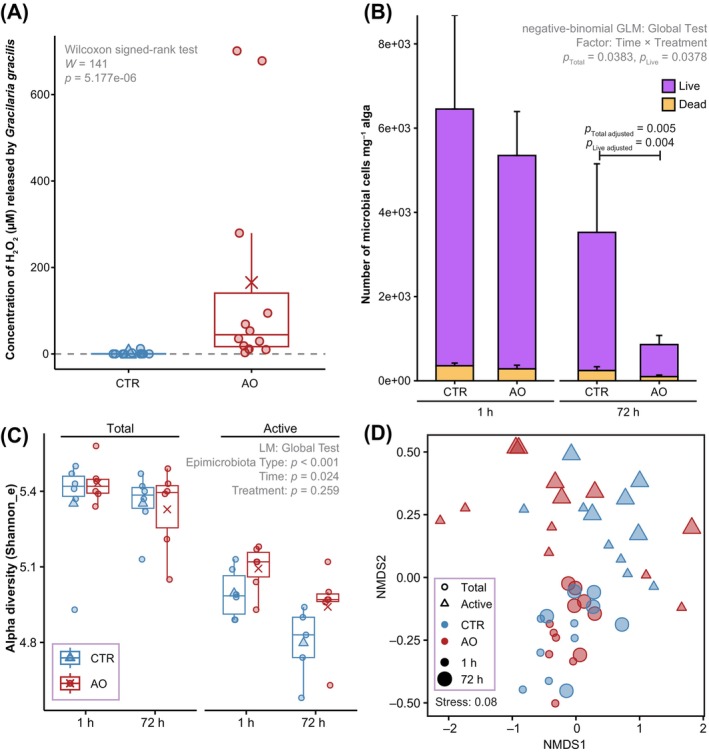
Effect of epimicrobiota type, time and immune elicitation on the abundance, diversity and structure of the *Gracilaria gracilis*‐associated epimicrobiota. (A) The concentration of H_2_O_2_ released by *G. gracilis* treated by either agar oligosaccharide (AO) or sterile deionized water as a control (CTR). (B) Numbers of live and dead microbial cells per milligram of *G. gracilis* (wet weight) at 1 h or 72 h after AO or CTR treatment. Bars represent the mean of six biological replicates, and error bars represent standard error. (C) Shannon index logged to the base of e representing the community alpha diversity. (D) Non‐metric multidimensional scaling (nMDS) plot of the Bray–Curtis dissimilarities of absolute amplicon sequencing variant abundance data. GLM, generalized linear model; LM, linear model.

Successful elicitation of the PTI in *G. gracilis* was demonstrated in response to AO. Specifically, we observed an increased release of H_2_O_2_ in *G. gracilis* treated with AO compared to the CTR (median: AO = 44.4 μM H_2_O_2_, CTR = 0.175 μM H_2_O_2_; Wilcoxon signed‐rank test, *W* = 141, *p* = 5.177e−06; Figure 2A). Live/dead staining showed that the *G. gracilis* epimicrobiota consisted predominately of living microbial cells (>90%) (Figures 2B and S1A,B). The total (live + dead) number and live microbial cells significantly decreased in AO‐treated samples in comparison to CTR samples at 72 h after treatment (multiple comparisons on negative‐binomial‐generalized linear model [GLM]: total: *z*‐value = 3.022, *p*_adjusted_ = 0.005; live: *z*‐value = 3.062, *p*_adjusted_ = 0.004; Figures 2B and S2 and Table S1), showing that the algal PTI elicitation removed microbial cells from the surface, but with a time lag. Our findings with AO elicitation are similar to what has previously been observed for direct pathogen elicitation, for example, a significant reduction of colony‐forming units (CFUs) for *Vibrio madracius* 72 h (but not earlier) after its inoculation onto the red seaweed *Laurencia dendroidea* (de Oliveira et al., 2017). However, our results contradict findings that the elicited immunity of *Gracilaria conferta* reduced up to 60% of bacterial CFUs already after 1 h (Weinberger & Friedlander, 2000). This difference suggests that the elicited immunity can have distinct temporal effects depending on the host species and the bacterial strain or community. In addition, immune elicitation of the kelp *Macrocystis pyrifera* using DAMPs (oligoguluronates) has been shown to afford protection against a subsequent infection by the alginolytic pathogen *Pseudomonas alginovora* (Küpper et al., 2002). Although the impact on the epiphytic microbiome abundance was not quantified, the resistance of *M. pyrifera* to lesions resulting from pathogen exposure was reduced by the NAD(P)H oxidase inhibitor diphenylene iodonium (DPI). DPI inhibits the algal oxidative burst response (Küpper et al., 2002) and has been shown to inhibit the oxidative burst in response to oligoagar in *G. gracilis* (Weinberger et al., 2010). Therefore, a future study that suppresses the seaweed oxidative burst with DPI and evaluates the seaweed phenotype and epimicrobiota abundance and composition could elucidate the direct role of NAD(P)H oxidase in seaweed PTI. Diseases in red seaweeds, including *Gracilaria* sp. (Lavilla‐Pitogo, 1992), *Kappaphycus alvarezii* and *Eucheuma denticulatum* (Largo et al., 1995), are generally characterized by an increase in bacterial abundances, likely resulting from an overgrowth of opportunistic bacteria (Kumar et al., 2016; Largo et al., 1995; Lavilla‐Pitogo, 1992). The reduction of microbial abundances due to elicited immunity may thus counteract such an overgrowth and mitigate associated impacts on host health.

The observed reduction in microbial cells after elicitation prompted us to further investigate whether the PTI of *G. gracilis* was affecting specific community members or if the effect was general in nature. To examine the effects on the total and active microbiome, we sequenced both the 16S rRNA genes and transcripts (Blazewicz et al., 2013). After rarefaction to the lowest number of sequences observed among all samples (i.e., 22,294) (Table S2 and Figure S3A,B), the relative abundance data were converted to absolute abundances using the numbers of 16S rRNA gene copies determined by qPCR (Figure S4A,B). We detected a significantly higher copy number of the 16S rRNA transcripts compared to the genes (negative‐binomial GLM, *z* = 2.838, *p* = 0.005; Table S3 and Figure S4). This is consistent with the observed high proportion (>90%) of living bacteria (Figures 2B and S1), which have been shown to have higher copy numbers of the 16S rRNA transcripts than the corresponding gene (Wang et al., 2023). While alpha diversity and community structure based on amplicon sequencing variants (ASVs) differed between total and active communities, there was no statistical support for differences between AO treatments and CTR (Figures 2C,D and S5 and Tables S4 and S5). These patterns were generally consistent when the ASV community data were also aggregated to different taxonomic levels, from species to phylum (Tables S4 and S5). Only one ASV (Zotu2954—GTDB taxonomy: *JAGFJK01* sp024102795; NCBI: *Pirellulaceae*) in the active microbiome (representing 0.003% of abundances of the active epimicrobiota) had significantly decreased abundances in the AO treatments compared to CTR after 72 h (multiple comparisons on negative‐binomial mGLM, *p*_adjusted_ = 0.044), demonstrating that in general the PTI did not cause a selection of specific phylotypes. This could be because the indigenous bacteria on *G. gracilis* have similar levels of adaptations to the innate immunity response allowing the community to maintain the same diversity and structure despite the loss in overall bacterial cell numbers. Ma et al. (2021) demonstrated that MAMP‐based PTI in *A. thaliana* influenced root microbiota structure variably, depending on the community composition. This underscores the role of MAMP‐based PTI in exerting selective pressure on root commensals, a specificity potentially attributed to an advanced co‐evolution state between the host and particular bacteria. However, to the best of our knowledge, our study represents the first study to directly investigate the effect of DAMP‐elicited immunity on the load and diversity of resident microbiota. Although it is yet unknown if PTI based on DAMP causes an even cell reduction in communities associated with terrestrial plants and/or other seaweed lineages, we postulate that a non‐selective reduction may serve as a mechanism to maintain microbial eubiosis of *G. gracilis* epimicrobiota. In situations where a danger‐response is triggered (e.g., cell or tissue damage), this would allow the seaweed to regulate bacterial overgrowth in a microbially rich aquatic environment, while still preserving the overall community diversity and structure. Thus, the current study provides a unique insight into the host innate immunity–microbe interaction in the seaweed holobiont.

## AUTHOR CONTRIBUTIONS

**Jiasui Li:** Conceptualization (equal); data curation (lead); formal analysis (lead); funding acquisition (equal); investigation (lead); methodology (lead); project administration (lead); software (lead); validation (lead); visualization (lead); writing – original draft (equal); writing – review and editing (equal). **Mahasweta Saha:** Investigation (supporting); supervision (supporting); writing – review and editing (equal). **Marwan E. Majzoub:** Formal analysis (supporting); software (supporting); validation (supporting); writing – review and editing (equal). **Teng Yang:** Software (supporting); validation (supporting); writing – review and editing (equal). **Haiyan Chu:** Resources (equal); writing – review and editing (equal). **Torsten Thomas:** Methodology (supporting); validation (supporting); writing – original draft (equal); writing – review and editing (equal). **Florian Weinberger:** Conceptualization (equal); data curation (supporting); funding acquisition (equal); investigation (supporting); methodology (equal); resources (equal); supervision (equal); writing – original draft (equal); writing – review and editing (equal). **Suhelen Egan:** Conceptualization (equal); funding acquisition (equal); methodology (equal); resources (equal); supervision (equal); writing – original draft (equal); writing – review and editing (equal).

## CONFLICT OF INTEREST STATEMENT

The authors declare no conflicts of interest.

## Supporting information


**Data S1.** Supporting information.

## Data Availability

The sequence data have been submitted to the BioProject database under accession number PRJNA642985. The scripts and data to reproduce all statistical analyses and visualization in this article are available at: https://doi.org/10.6084/m9.figshare.24412672.
