# Supplementary material for: Non‐selective microbiota reduction after the elicitation of a seaweed's immune response
Source: Environ Microbiol Rep. 2024 May 17;16(3):e13268. doi: 10.1111/1758-2229.13268 (PMC11101764; doi:10.1111/1758-2229.13268)
Supplement: Supplementary file 1 — Data S1. Supporting information. [file EMI4-16-e13268-s001.zip › Supporting information.docx]

**Supporting Information**

**Non-selective microbiota reduction after the elicitation of a seaweed’s immune response**

Jiasui Li^1,2^, Mahasweta Saha^3,4^, Marwan E. Majzoub^2,5^, Teng Yang^1^, Haiyan Chu^1,6^, Torsten Thomas^2^, Florian Weinberger^3^, and Suhelen Egan^2^

^1^ State Key Laboratory of Soil and Sustainable Agriculture, Institute of Soil Science, Chinese Academy of Sciences, Nanjing 210008, China

^2^ Centre for Marine Science and Innovation, School of Biological, Earth and Environmental Sciences, Faculty of Science, The University of New South Wales, Kensington, NSW, 2052, Australia

^3^ Marine Ecology Division, GEOMAR Helmholtz Centre for Ocean Research Kiel, Wischhofstrasse 1-3, 24148 Kiel, Germany

^4^ Marine Ecology and Biodiversity, Plymouth Marine Laboratory, Prospect Place, Plymouth PL1 3DH, UK

^5^ School of Biomedical Sciences, Faculty of Medicine and Health, The University of New South Wales, Kensington, NSW, 2052, Australia

^6^ University of Chinese Academy of Sciences, Beijing 100049, China

**Corresponding Author**

Suhelen Egan,

Centre for Marine Science and Innovation, School of Biological, Earth and Environmental Sciences, Faculty of Science, The University of New South Wales, Kensington, NSW, 2052, Australia

(+61) 2 9385 8569

s.egan@unsw.edu.au

**Co-corresponding Authors**

Florian Weinberger,

Marine Ecology Division, GEOMAR Helmholtz Centre for Ocean Research Kiel, Wischhofstrasse 1-3, 24148 Kiel, Germany

(+49) 431 600 4516

fweinberger@geomar.de

Jiasui Li,

State Key Laboratory of Soil and Sustainable Agriculture, Institute of Soil Science, Chinese Academy of Sciences, Nanjing 210008, China

(+86) 185 8185 4283

jiasuili@issas.ac.cn

**Contents:**

**Supplementary Experimental Procedures:**

**Collection and maintenance of *Gracilaria gracilis***

*G. gracilis* samples were collected from the shore of the Ile de Batz (48°44'54’N, 3°59'47’W) (Brittany, France) on the 19^th^ of January 2019 and transported to the laboratories of the GEOMAR Helmholtz Centre for Ocean Research (Kiel, Germany) within 20 hours. Samples were routinely kept at 15 ± 1°C under a day-night cycle of 15 h: 9 h (photon flux density for the day: 75 µmol m^-2^ s^-1^) for the duration of the study. *G. gracilis* samples were maintained in Baltic Sea seawater (salinity adjusted to 33 practical salinity units) for one month to acclimatise before being used for experiments.

**Preparation of agar oligosaccharides**

Low-gelling temperature agarose (1% w/v, Invitrogen, Darmstadt, Germany) was dissolved in 500 mL of boiling deionised water and cooled down to 39°C under constant stirring. *β*-agarase (10,000 enzyme units, Sigma-Aldrich, Darmstadt, Germany) was added and the mixture was incubated for 6 h. The mixture was boiled for five minutes to stop the reaction and approximately 90% of its water content was removed by rotary evaporation using a Büchi rotavapor R3 (Essen, Germany). To separate AO from unhydrolyzed agarose the condensed mixture was dialysed three times overnight in 5 L of deionised water using a dialysis tube with a SpectraPor MWCO of 2000 (Repligen, Ravensburg, Germany). Between successive dialysis steps the retentate was condensed to a volume of 50 mL. The permeate, containing AO, of all three dialysis steps was pooled, condensed by rotary evaporation, and freeze-dried. The material was resuspended in deionised water and the molarity of AO was determined based on the content of reducing saccharides following the protocol described in (Weinberger et al., 2005). A 20 mM stock solution of AO was then stored at -20°C.

**Elicitation of *G. gracilis* immune response**

Algal fragments (ca. 2 g) taken from random individuals were rinsed in 0.22 µm filtered seawater (FSW) to remove loosely attached microorganisms, and then transferred to separate aquaria containing 800 ml FSW and left overnight to acclimatise. The water in each aquarium was replaced with 160 ml fresh FSW, which was supplemented with either 800 µl of 20 mM AO or the same amount of sterile deionised water (as a control - CTR). After 1 h, the AO was removed by replacing the water in each aquarium with 800 ml fresh FSW (the same procedure was also applied to the controls). Samples (*n* = 6 for each treatment) were collected, and after 72 h, the remaining samples (*n* = 6 for each treatment) were collected. From each sample ca. 0.5 g tissue was immediately processed for the quantification of microbiota cell numbers using epifluorescence microscopy, and 0.5 g tissue was immediately frozen in liquid nitrogen and then stored at -80°C for further DNA and RNA extractions.

**Quantification of hydrogen peroxide**

Hydrogen peroxide (H_2_O_2_) levels in the medium of algal samples were measured prior to and 15 min after the AO or CTR treatment using a luminol/ferricyanide assay with a Turner Design 20/20 luminometer as previously described (Weinberger et al., 2005). A standard curve was established (log-linear interpolation formula: *y* = 1.0851*x* + 0.7558, *r*^2^ = 0.999) by measuring 10-fold serial dilutions of 30% H_2_O_2_.

**Quantification of live and dead bacteria on *G. gracilis***

Epiphytic cells associated with *G. gracilis* were quantified using a method adapted from (Saha et al., 2016). Algal tissue (0.5 g) was vortexed with 15 sterile glass beads (Ø = 2 mm) in 50 ml FSW for 30 s, followed by vacuum filtration of 5 ml onto polycarbonate filters (black, pore size Ø = 0.2 µm, filter size Ø = 25 mm, DHI Hörsholm, Denmark). The filters were stained with the LIVE/DEAD™ BacLight™ Bacterial Viability Kit following the manufacturer’s instructions. The stained filters were assessed by epifluorescence microscopy with a magnification of 400× (Zeiss Axioscope.A1 microscope equipped with a Jenoptik ProgRes camera system) (Bouchez et al., 2001, Grubwieser et al., 2022), using an epifluorescence filter configuration of 470 nm for excitation, and integrated of the green (510-540 nm) and red (620-650 nm) for emission. For each filter, 10 photos were taken from randomly chosen fields of view (435.2 µm × 327.68 µm). Live and dead microbial numbers were estimated by counting spherical fluorescent particles with a diameter of 0.25-1.0 µm with green (live cells) and yellow/orange/red (cell-membrane damaged and dead cells) (DeLeon-Rodriguez et al., 2013) colour using the *Analyse Particles* function in ImageJ (Schindelin et al., 2012).

**DNA, RNA extraction and synthesis of cDNA**

To describe the bacterial community associated with *G. gracilis* both DNA and RNA were extracted for each algal sample. Extracted DNA and RNA were subsequently used for qPCR (or RT-qPCR) and amplicon sequencing analyses to assess the total and active microbial communities, respectively.

Frozen algal tissues (0.5 g) were homogenised in 100 μl RNAse free TE buffer, pH 7.4 (Thermo Fisher Scientific, Altrincham, UK) and divided equally for DNA and RNA extractions. DNA was extracted using the Quick-DNA Fecal/Soil Microbe Kits (Zymo Research, Irvine, CA, USA) following the manufacturer’s instructions. The RNA was extracted using a TRIzol-based protocol. Briefly, a TRIzol RNA isolation reagent (Thermo Fisher Scientific, San Diego, CA, USA) was added to frozen samples on ice, the homogenate was transferred to a Lysing Matrix E (MP Biomedicals, Eschwege, Germany) bead tube and vortexed to lyse cells. The lysate was transferred to a 1.5 mL centrifuge tube and vortexed for 30 s twice. Following a 5 min incubation at room temperature, 200 μl chloroform was added to separate the RNA phase. The RNA was precipitated with 600 μl cold isopropanol and the pellet was washed twice with 75% ethanol. The DNA in RNA extracts was removed using RQ1 RNase-Free DNase (Promega, Alexandria, Australia) following the manufacturer’s instructions. The DNA-free RNA was re-purified with cold sodium acetate (pH 5.2), isopropanol and 75% ethanol. The DNA and RNA concentrations were then determined by Qubit® 3.0 fluorometer (Thermo Fisher Scientific, Scoresby, Australia). The RNA was used to synthesise cDNA with the ProtoScript® II First Strand cDNA Synthesis Kit (NEW ENGLAND BioLabs, Notting Hill, Australia) and a Randomised Primer Mix following the manufacturer’s instructions.

**Quantification of total and active epimicrobiota of *G. gracilis***

To determine the absolute abundance of the 16S rRNA gene, qPCR of the hypervariable V3-V4 regions (ca. 500 bp) of the 16S rRNA gene and cDNA were performed using the primers 341F (5’-CCTACGGGNGGCWGCAG-3’) and 785R (5’-GACTACHVGGGTATCTAATCC-3’) (Klindworth et al., 2013) using methods adapted from (Nappi et al., 2023). The pPNA (5’-GGCTCAACCCTGGACAG-3’) and mPNA (5’-GGCAAGTGTTCTTCGGA-3’) clamps were used to suppress the amplification of plastid and mitochondrial DNA from the host algae (Lundberg et al., 2013). The PCR mixture contained 5 µl of PerfeCTa SYBR FastMix (Quantabio, Beverly, MA, USA), 0.3 µl (10 µM) of each 341F/785R primer, 0.3 µl (20 µM) of each PNA oligonucleotides, 2.8 µl of Ambion® nuclease-free water and 1 µl of DNA or cDNA template for a total volume of 10 µl per sample. The PCR cycling consisted of an initial denaturation at 95°C for 5 min, 40 cycles of denaturation at 95°C for 15 s, PNA oligonucleotides binding at 78°C for 10 s, primers 341F/785R annealing at 55°C for 45 s and extension at 72°C for 45 s, followed by melt curve analysis from 55°C to 95°C at 0.5°C intervals. The Mic qPCR Cycler (BioMolecular Systems, Upper Coomera, Australia) and micPCR software (version 2.6.3) were used for amplification and analysis. The cycle threshold was automatically set by the software. The Dynamic method was used for the baseline correction, determination of C_q_ values and efficiency for individual samples (Ruijter et al., 2009). To quantify the 16S rRNA gene and transcript numbers, a standard curve (*y* = - 3.704*x* + 39.077, *r*^2^ = 0.994, *efficiency* = 0.86) was established using 10-fold serial dilutions of a mock bacterial community DNA standard (ZymoBIOMICS^TM^, Zymo Research) (Fig. S3). For each sample, at least three technical replicates were included. Reactions were repeated for any technical replicates with a C_q_ variance > 0.5 cycles or efficiency < 0.85 or > 1.1. The copy number of 16S rRNA gene or transcript per gram algal tissue for each DNA/RNA sample was calculated based on the 16S rRNA gene/transcript copy numbers quantified by qPCR, the dilution factor and the algal tissue biomass used for DNA/RNA extraction.

**16S rRNA gene amplicon library preparation, sequencing, and data processing**

The DNA- and cDNA -based 16S rRNA gene amplicon libraries were prepared using methods adapted from (Li et al., 2022). Briefly, the hypervariable V3-V4 regions of the bacterial 16S rRNA gene and transcript were amplified using the 341F/785R primers, with the addition of the abovementioned pPNA and mPNA clamps. PCR amplicons were quantified using gel electrophoresis. Paired-end sequencing (2×300 bp) of the resulting amplicons was performed at the Ramaciotti Centre for Genomics (UNSW, Australia) on an Illumina MiSeq platform as per the MiSeq System User Guide (Kozich et al., 2013). Raw sequencing reads were processed using TRIMMOMATIC version 0.36 (Bolger et al., 2014) and USEARCH version 11.0.667 (Edgar, 2010) following the procedure described in (Wemheuer and Wemheuer, 2017). Briefly, filtered high-quality sequences were denoised and clustered into amplicon sequence variants (ASVs) (Prodan et al., 2020) using the UNOISE algorithm implemented in USEARCH. Chimeric sequences were removed with UCHIME (Edgar et al., 2011) *de novo* during ASV clustering and subsequently with a reference-based comparison against the SILVA v138 database (Yilmaz et al., 2014). The non-chimeric sequences were taxonomically classified using the BLCA (Gao et al., 2017) against the GTDB r214 database (Parks et al., 2022). The processed sequences were mapped onto ASV sequences to calculate the count distribution of each ASV in every sample. As the GTDB database only includes prokaryotic sequences, the sequences of unclassified ASVs were further searched against the SILVA v138 database (Yilmaz et al., 2014), the metaPR^2^ database version 5.0.0 (Vaulot et al., 2022) and a database (see Data Availability) prepared with the chloroplast and mitochondrion sequences of *G. gracilis* (Lipinska et al., 2023). A similarity cut-off of 94% (Lundberg et al., 2013) and BioSAK v1.69.4 (https://github.com/songweizhi/BioSAK) and BLAST 2.13.0+ (McGinnis and Madden, 2004) were used for the search. The ASVs with their best match (based on the bitscore) hitting any non-bacterial taxa (i.e., “Eukaryota” in metaPR^2^, “Mitochondria” or “Chloroplast” in SILVA, and “*Gracilaria*” database) were annotated as non-bacteria. 17 ASVs representing 0.67% and 9.02% of the relative read abundance in the DNA-based and of cDNA-based amplicon datasets, respectively, were found to be derived from *G. gracilis*’ chloroplast or mitochondrion. All these non-bacterial and singleton ASVs were removed, and the corresponding portions were also removed from qPCR data. The sequencing depth was visualised using rarefaction curves and the sampling efficiency was estimated with Good’s coverage indices using the R package vegan (Oksanen et al., 2019) and QsRutils (Zhang et al., 2017). To account for the uneven sequencing depth, count data were subsampled to the lowest reads observed among samples using USEARCH. The qPCR data were used to transform the relative read abundances of bacterial ASVs into absolute abundance data (Morton et al., 2019), which was used for all subsequent analysis.

**Statistical analysis**

All statistical analyses were performed in R version 4.0.3 (R Core Team, 2019). A non-parametric Wilcoxon signed-rank test was performed to compare the release of H_2_O_2_ between AO and CTR treatments using the *wilcox.test* function in R. The count data of total and live bacterial cell numbers (per mg algal tissue) and 16S rRNA gene/transcript copies were fitted to Generalised Linear Models (GLM) assuming a negative binomial distribution using the *glm.nb* function of the MASS package (Venables and Ripley, 2013). The effects of ‘Time’ (two levels: 1 h and 72 h), ‘Treatment’ (two levels: AO and CTR), and their interactions were assessed using the *summary* function of the base R package. Community alpha diversity indices, i.e., Shannon logged to base e (Shannon_e) for diversity, Chao1 for richness, 1-Berger_Parker for evenness for the total and active epimicrobiota were calculated using USEARCH and subsequently fitted to linear models (LM), and an analysis of variance (ANOVA) on the aforementioned factors were performed; the *lm* and *anova* functions in the R package were used for model fitting and *p*-value calculation, respectively. The absolute abundances (i.e., 16S rRNA gene or transcript copy numbers) of each ASV was fitted to multivariate GLM (mGLM) assuming a negative binomial distribution (Wang et al., 2012) and ANOVA was performed on the mGLMs. The global test *p*-values and *p*-values for univariate GLM tests were calculated via 999 parametric bootstraps. For multiple comparisons, the *p* values were adjusted using an FDR (false discovery rate) method (Benjamini and Hochberg, 1995). For all hypothesis tests in this study, the *p*-value of <0.05 was considered significant.

**Supplementary Figures** (Figure S1-S5)


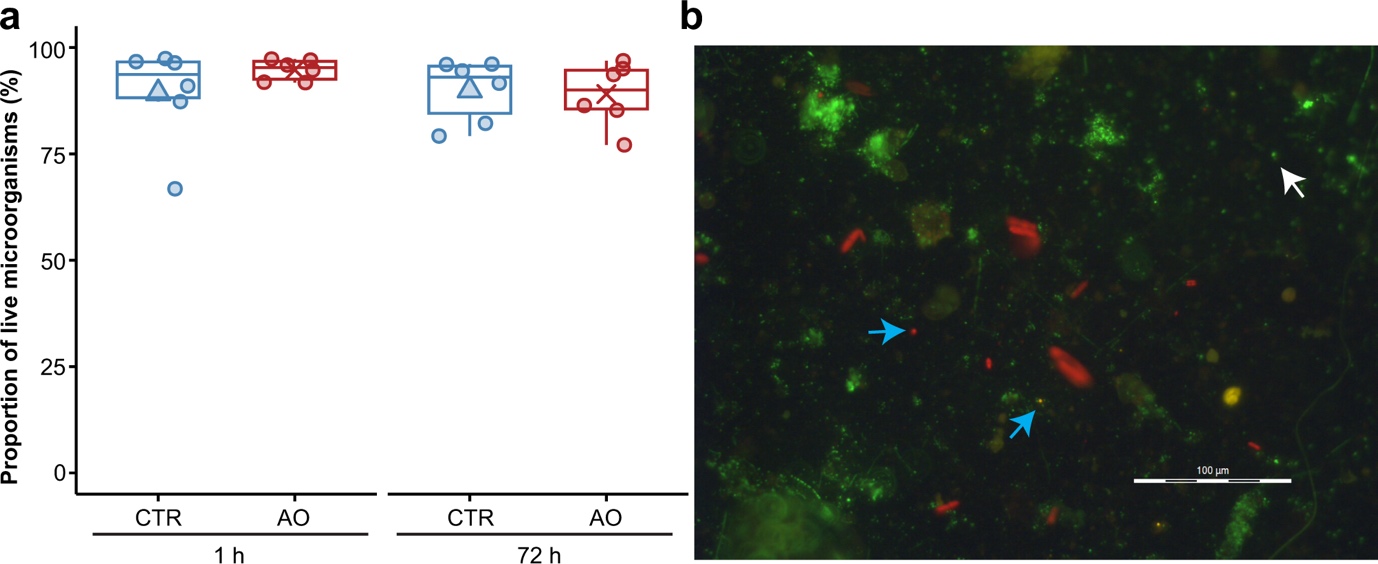


**Figure** **S1.** Microorganisms associated with *Gracilaria gracilis*. **(a)** The proportion of live in total (live + dead) microorganisms of different treatments and times. The lower and upper hinges of boxplots show the 25^th^ and 75^th^ percentiles, respectively. The boxplot whiskers extend to 1.5 IQR (inter-quartile range) to the hinges, and the middle line represents the median calculated on *n* = 6 biological replicates, which are shown as overlayed points. The mean values calculated for each treatment or at each time point are shown as either a triangle (CTR) or cross (AO). **(b)** A representative epifluorescence microscopy image. The cell quantification was based on staining with LIVE/DEAD™ BacLight™ Bacterial Viability Kit which stains live cells green (white arrow) and dead (membrane-damaged) cells orange-red (blue arrow).


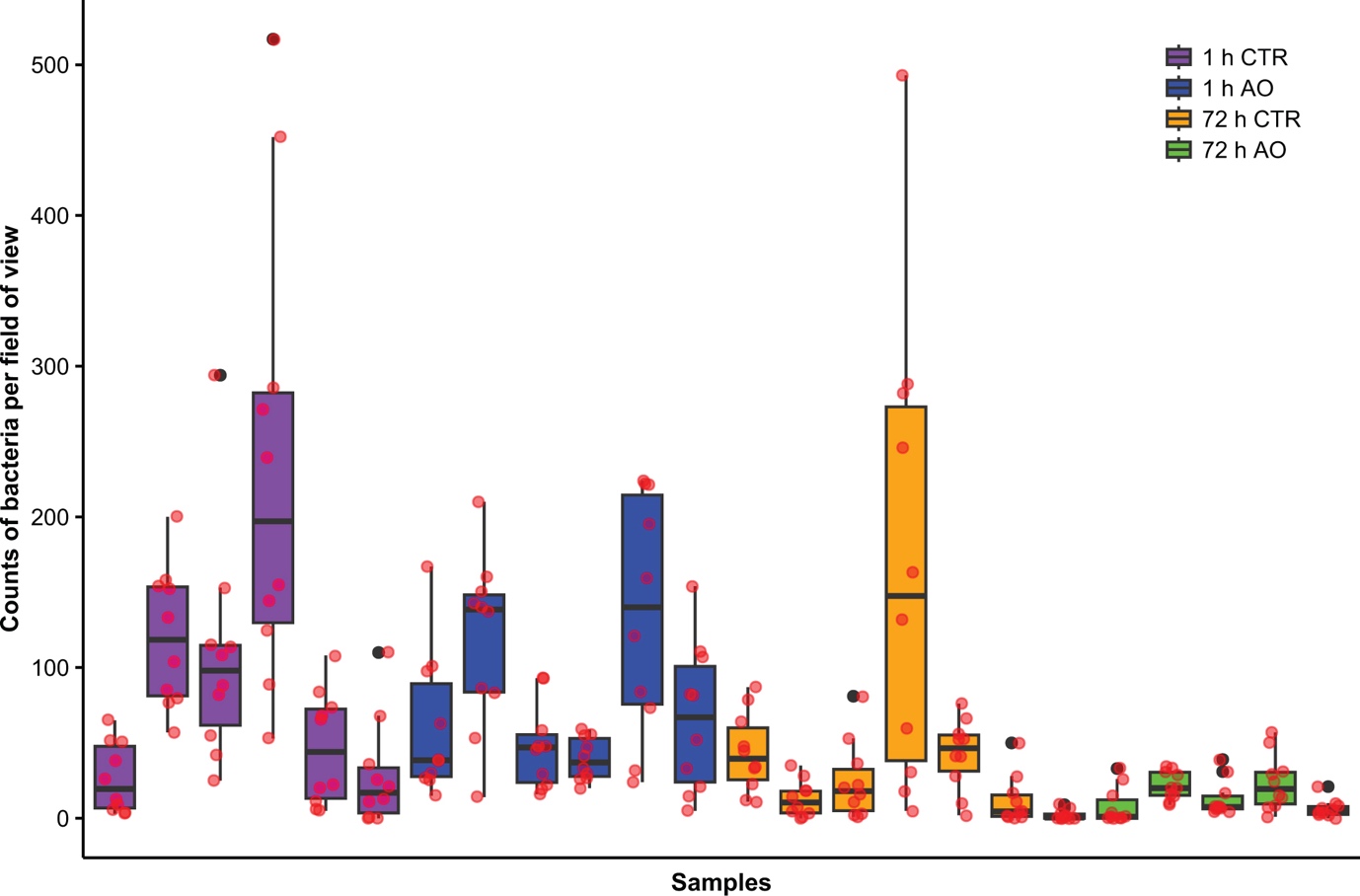


**Figure S2.** Bacterial cell counts in individual filter samples. Each boxplot represents an individual filter sample, and each red dot overlayed represents one replicate field of view. For each sample, 10 technical replicates (fields of view) are examined.


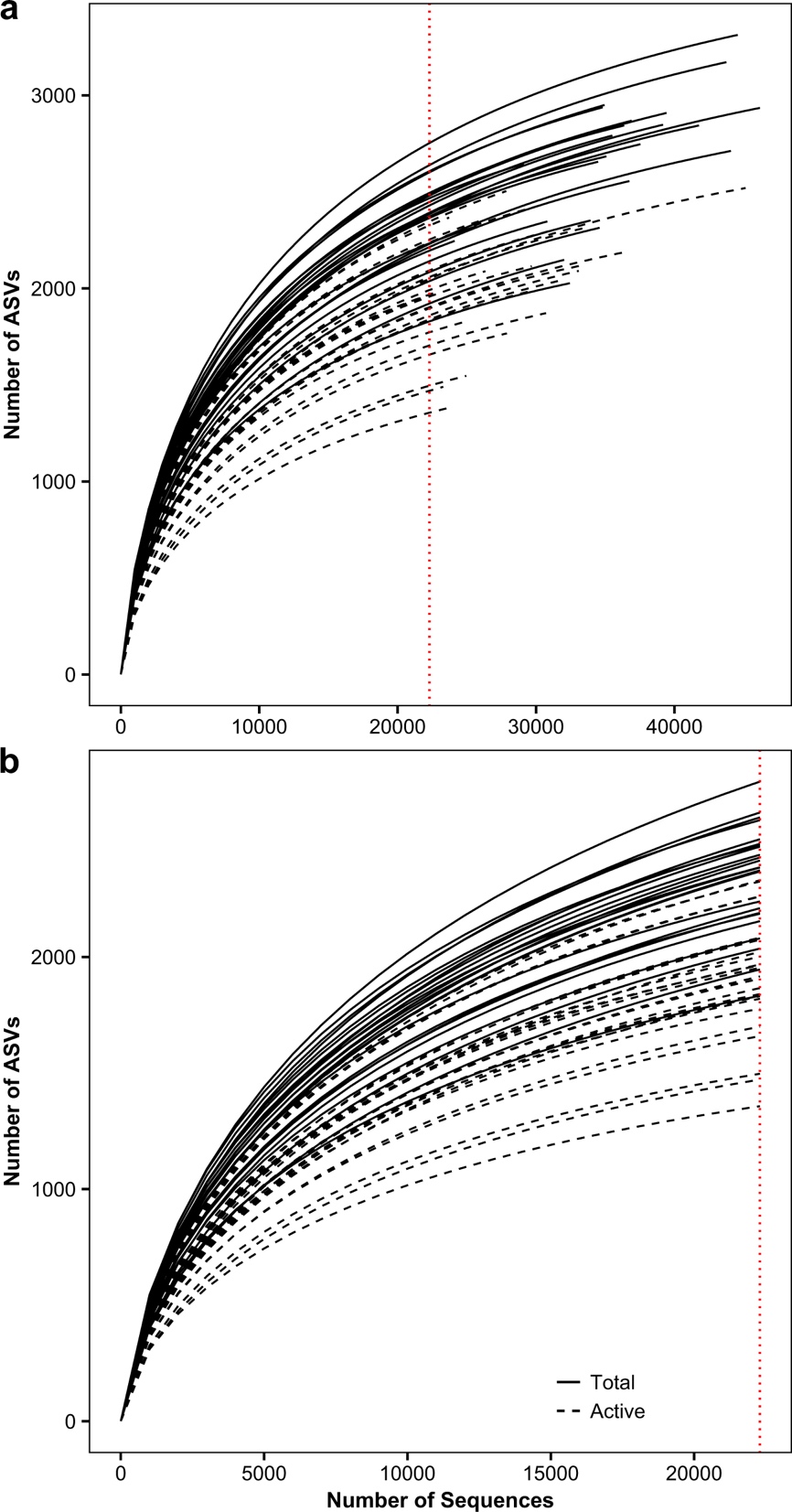


**Figure S3.** Rarefaction curves. The bacterial ASV numbers are plotted against the numbers of quality-filtered sequences before **(a)** and after **(b)** being subsampled to the lowest sequences yielded (the vertical dot lines show 22,294) for the total and active bacterial communities of *G. gracilis*.

**
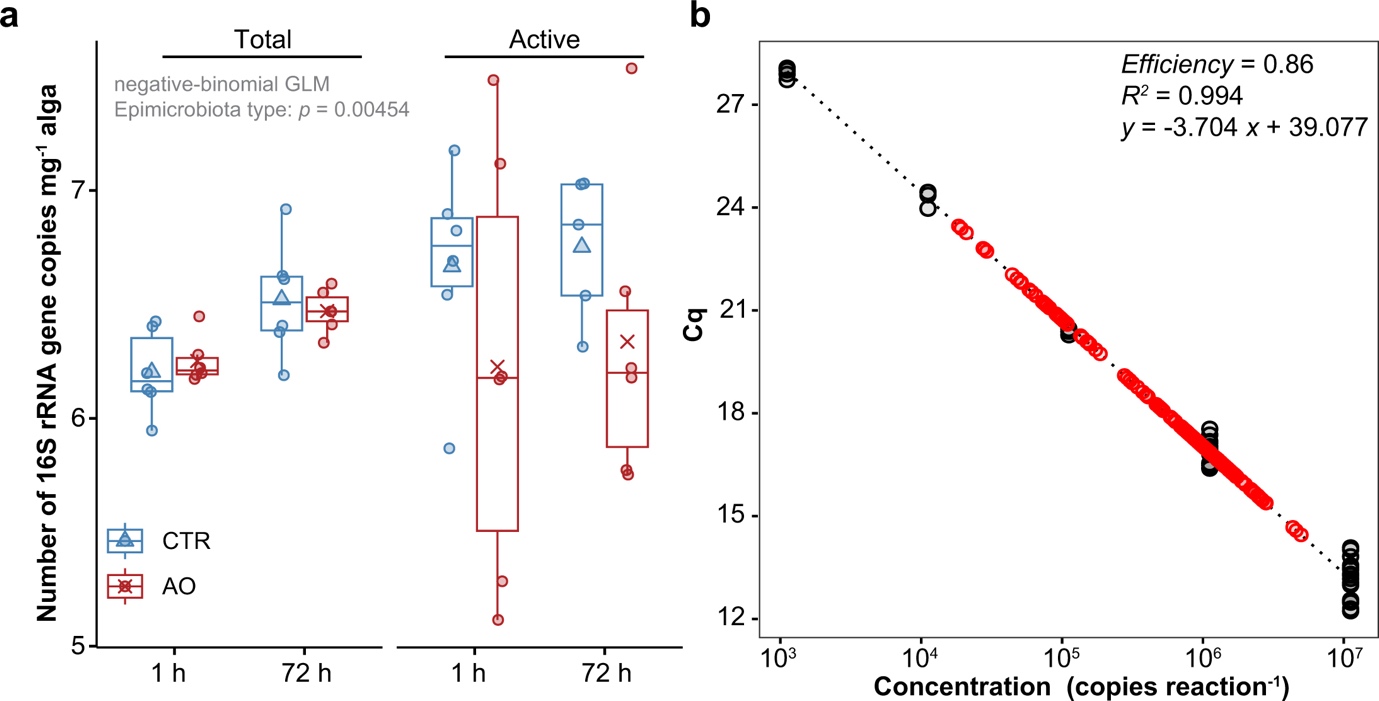
**

**Figure S4.** The number of 16S rRNA gene and transcript copies of the *G. gracilis* epimicrobiota. **(a)** Log of the copy numbers of epimicrobiota types (total or active), treatments (AO or CTR), and time of incubation (1 h or 72 h). **(b)** The standard curve of copy numbers of the 16S rRNA gene based on a qPCR analysis of 10-fold serial dilutions of the 16S rRNA gene of a mock bacterial community DNA standard (ZymoBIOMICS^TM^). The standards are shown as black dots and samples are shown as red dots. For each serial dilution of the standards or the bacterial community samples, at least three technical replicates with *efficiency* >0.85 and <1.1 were obtained.

**
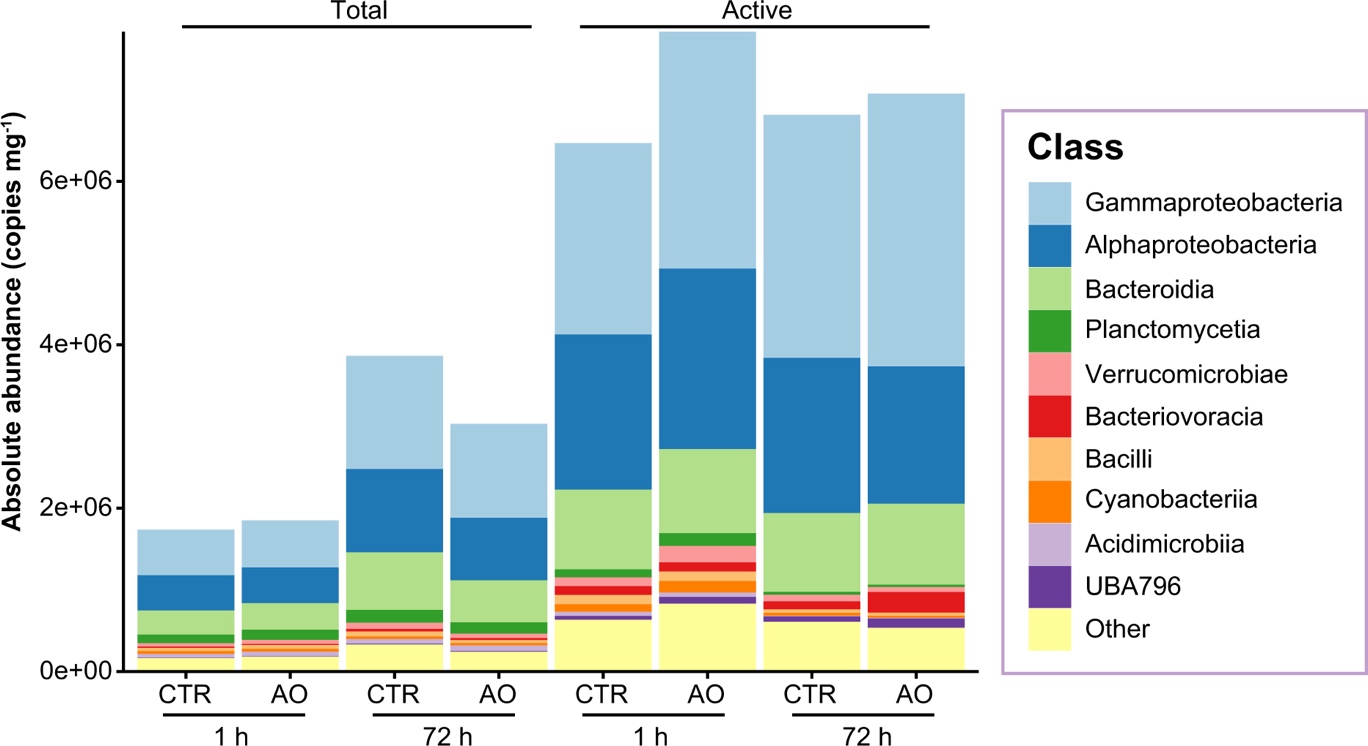
**

**Figure S5.** Taxonomical composition of *G. gracilis* epimicrobiota based on the absolute abundance (copy numbers of 16S rRNA gene or transcript per mg fresh algae) of bacterial classes.

**Supplementary Tables** (Table S1-S5)

**Table S1.** Wald tests on the effect of elicited immune response on the number of total (live + dead) and live microbial cells in *G. gracilis* epimicrobiota. The epifluorescence microscopy generated counts are fitted into a Generalised Linear Model (GLM) assuming a negative-binomial distribution using the *glm.nb* function in the R package MASS. The effects of ‘Time’ (two levels: 1 h and 72 h), ‘Treatment’ (two levels: AO and CTR), and their interaction are assessed using the *summary* function of the base R package. Post-hoc tests were performed to assess the effect of treatment within each time point. (Excel data file)

**Table S2.** General characteristics of the 16S rRNA amplicon sequencing data of total (gene-based) and active (transcript-based) bacterial communities associated with *G. gracilis* treated by either agar oligosaccharides (AO) or sterile deionised water as control (CTR), collected at 1 h or 72 h post-elicitation. For each treatment at each time point, six biological replicates were included. (Excel data file)

**Table S3.** The effect of epimicrobiota type, time, and treatment on the 16S rRNA gene/transcript copy numbers of *G. gracilis*-associated epimicrobiota. The copy numbers determined by qPCR normalised to per mg fresh weight algae were fitted into a negative-binomial GLM using the *glm.nb* function in R package MASS, and the ‘Epimicrobiota Type’ (two levels: Total and Active), ‘Time’ (two levels: 1 h and 72 h), and ‘Treatment’ (two levels: CTR and AO) were fitted sequentially and as interactive factors. The *p* values were calculated using the *summary* function in the base R package.

| **Factor** | ***Standard Error*** | ***z value*** | ***p*** |
| --- | --- | --- | --- |
| Epimicrobiota Type | 0.5120 | 2.838 | 0.00454 |
| Time | 0.5120 | 0.204 | 0.83832 |
| Treatment | 0.5120 | 0.380 | 0.70417 |
| Epimicrobiota Type×Time | 0.7241 | 0.832 | 0.40523 |
| Epimicrobiota Type×Treatment | 0.7241 | 0.180 | 0.85735 |
| Time×Treatment | 0.7420 | 0.212 | 0.83240 |
| Epimicrobiota Type×Time×Treatment | 1.0368 | 0.146 | 0.88430 |

**Table S4.** Analysis of variance (ANOVA) assessing the effect of epimicrobiota type, time, and treatment on the community diversity (represented by Shannon_e indices), richness (Chao1 indices), and evenness (1-Berger-Parker) of *G. gracilis*-associated epimicrobiota at different phylogenetic levels (i.e., from ASV to phylum). The alpha diversity indices were fitted into linear models (LM) where the ‘Epimicrobiota Type’, ‘Time’, and ‘Treatment’ were fitted sequentially and as interactive factors. The *lm* and *anova* functions in the stats R package were used for model fitting and *p*-value calculation, respectively. Multiple comparisons were performed to assess the effect of treatment within each time point and in each epimicrobiota type. For multiple comparisons, the *p* values were adjusted using an FDR method. (Excel data file)

**Table S5.** ANOVA tests on the effect of epimicrobiota type, time, treatment, and their interactions on the community structure at ASV and different taxonomic levels (i.e., from species to phylum). The absolute abundances of ASVs or other phylotypes were fitted into multivariate generalised linear models (mGLM) assuming a negative binomial distribution, wherein each ASV or phylotype was treated as a GLM. The ‘Epimicrobiota Type’ (Total or Active), ‘Treatment’ (AO or CTR), and ‘Time’ (1 h or 72 h) were fitted as interactive factors. Multiple comparisons were performed to assess the effect of treatment within each time point and in each epimicrobiota type. For multiple comparisons, the *p* values were adjusted using an FDR method. For univariate GLM tests the *p* values were adjusted using a step-down resampling procedure with 999 resamplings. (Excel data file)

**References**

Benjamini, Y. & Hochberg, Y. 1995. Controlling the false discovery rate: A practical and powerful approach to multiple testing. *Journal of the Royal Statistical Society: Series B (Methodological),* 57**,** 289-300. Available from: <https://doi.org/10.1016/s0166-4328(01)00297-2>

Bolger, A. M., Lohse, M. & Usadel, B. 2014. Trimmomatic: a flexible trimmer for Illumina sequence data. *Bioinformatics,* 30**,** 2114-2120. Available from: <https://doi.org/10.1093/bioinformatics/btu170>

Bouchez T, Dabert P, Wagner M, Godon J-J & Moletta R. 2001. Quantification of bacterial populations in complex ecosystems using fluorescent in situ hybridization, confocal laser scanning microscopy and image analysis. *Genetics, Selection, Evolution,* 33: S307-S318. Available from: <https://doi.org/10.1186/BF03500886>

DeLeon-Rodriguez, N., Lathem, T. L., Rodriguez, R. L., Barazesh, J. M., Anderson, B. E., Beyersdorf, A. J. et al. 2013. Microbiome of the upper troposphere: species composition and prevalence, effects of tropical storms, and atmospheric implications. *Proceedings of the National Academy of Sciences of the United States of America,* 110**,** 2575-80. Available from: <https://doi.org/10.1073/pnas.1212089110>

Edgar, R. C. 2010. Search and clustering orders of magnitude faster than BLAST. *Bioinformatics,* 26**,** 2460-2461. Available from: <https://doi.org/10.1093/bioinformatics/btq461>

Edgar, R. C., Haas, B. J., Clemente, J. C., Quince, C. & Knight, R. 2011. UCHIME improves sensitivity and speed of chimera detection. *Bioinformatics,* 27**,** 2194-2200. Available from: <https://doi.org/10.1093/bioinformatics/btr381>

Gao, X., Lin, H., Revanna, K. & Dong, Q. 2017. A Bayesian taxonomic classification method for 16S rRNA gene sequences with improved species-level accuracy. *BMC Bioinformatics,* 18**,** 247. Available from: <https://doi.org/10.1186/s12859-017-1670-4>

Grubwieser P, Hoffmann A, Hilbe R, Seifert M, Sonnweber T, Böck N, Theurl I, Weiss G & Nairz M. 2022. Airway epithelial cells differentially adapt their iron metabolism to infection with *Klebsiella pneumoniae* and *Escherichia coli* *in vitro*. *Frontiers in Cellular and Infection Microbiology*, 12. Available from: <https://doi.org/10.3389/fcimb.2022.875543>

Klindworth, A., Pruesse, E., Schweer, T., Peplies, J., Quast, C., Horn, M. & Glöckner, F. O. 2013. Evaluation of general 16S ribosomal RNA gene PCR primers for classical and next-generation sequencing-based diversity studies. *Nucleic Acids Research,* 41**,** e1-e1. Available from: <https://doi.org/10.1093/nar/gks808>

Kozich, J. J., Westcott, S. L., Baxter, N. T., Highlander, S. K. & Schloss, P. D. 2013. Development of a dual-index sequencing strategy and curation pipeline for analyzing amplicon sequence data on the MiSeq Illumina sequencing platform. *Applied and Environmental Microbiology,* 79**,** 5112-20. Available from: <https://doi.org/10.1128/AEM.01043-13>

Li, J., Majzoub, M. E., Marzinelli, E. M., Dai, Z., Thomas, T. & Egan, S. 2022. Bacterial controlled mitigation of dysbiosis in a seaweed disease. *The* *ISME Journal,* 16**,** 378-387. Available from: <https://doi.org/10.1038/s41396-021-01070-1>

Lipinska, A. P., Krueger-Hadfield, S. A., Godfroy, O., Dittami, S. M., Ayres-Ostrock, L., Bonthond, G. et al. 2023. The rhodoexplorer platform for red algal genomics and whole-genome assemblies for several *Gracilaria* species. *Genome Biology and Evolution,* 15**,** evad124. Available from: <https://doi.org/10.1093/gbe/evad124>

Lundberg, D. S., Yourstone, S., Mieczkowski, P., Jones, C. D. & Dangl, J. L. 2013. Practical innovations for high-throughput amplicon sequencing. *Nature Methods,* 10**,** 999-1002. Available from: <https://doi.org/10.1038/nmeth.2634>

McGinnis, S. & Madden, T. L. 2004. BLAST: at the core of a powerful and diverse set of sequence analysis tools. *Nucleic Acids Research,* 32**,** W20-W25. Available from: <https://doi.org/10.1093/nar/gkh435>

Morton, J. T., Marotz, C., Washburne, A., Silverman, J., Zaramela, L. S., Edlund, A. et al. 2019. Establishing microbial composition measurement standards with reference frames. *Nature Communications,* 10**,** 2719. Available from: <https://doi.org/10.1038/s41467-019-10656-5>

Nappi, J., Goncalves, P., Khan, T., Majzoub, M. E., Grobler, A. S., Marzinelli, E. M. et al. 2023. Differential priority effects impact taxonomy and functionality of host-associated microbiomes. *Molecular Ecology,* 32**,** 6278-6293. Available from: <https://doi.org/10.1111/mec.16336>

Oksanen, J., Blanchet, F. G., Friendly, M., Kindt, R., Legendre, P., McGlinn, D. et al. 2019. Package ‘vegan’. *Community ecology package, version,* 2. Available from: <https://github.com/vegandevs/vegan>

Parks, D. H., Chuvochina, M., Rinke, C., Mussig, A. J., Chaumeil, P.-A. & Hugenholtz, P. 2022. GTDB: an ongoing census of bacterial and archaeal diversity through a phylogenetically consistent, rank normalized and complete genome-based taxonomy. *Nucleic Acids Research,* 50**,** D785-D794. Available from: <https://doi.org/10.1093/nar/gkab776>

Prodan, A., Tremaroli, V., Brolin, H., Zwinderman, A. H., Nieuwdorp, M. & Levin, E. 2020. Comparing bioinformatic pipelines for microbial 16S rRNA amplicon sequencing. *PLOS One,* 15**,** e0227434. Available from: <https://doi.org/10.1371/journal.pone.0227434>

Ruijter, J. M., Ramakers, C., Hoogaars, W. M., Karlen, Y., Bakker, O., van den Hoff, M. J. et al. 2009. Amplification efficiency: linking baseline and bias in the analysis of quantitative PCR data. *Nucleic Acids Research,* 37**,** e45. Available from: <https://doi.org/10.1093/nar/gkp045>

Saha, M., Wiese, J., Weinberger, F. & Wahl, M. 2016. Rapid adaptation to controlling new microbial epibionts in the invaded range promotes invasiveness of an exotic seaweed. *Journal of Ecology,* 104**,** 969-978. Available from: <https://doi.org/10.1111/1365-2745.12590>

Schindelin, J., Arganda-Carreras, I., Frise, E., Kaynig, V., Longair, M., Pietzsch, T. et al. 2012. Fiji: an open-source platform for biological-image analysis. *Nature Methods,* 9**,** 676-682. Available from: <https://doi.org/10.1038/nmeth.2019>

Vaulot, D., Sim, C. W. H., Ong, D., Teo, B., Biwer, C., Jamy, M. et al. 2022. metaPR2: A database of eukaryotic 18S rRNA metabarcodes with an emphasis on protists. *Molecular Ecology Resources,* 22**,** 3188-3201. Available from: <https://doi.org/10.1111/1755-0998.13674>

Venables, W. N. & Ripley, B. D. 2013. *Modern applied statistics with S-PLUS*, Springer Science & Business Media. Available from: <https://doi.org/10.1007/978-0-387-21706-2>

Wang, Y., Naumann, U., Wright, S. T. & Warton, D. I. 2012. mvabund– an R package for model-based analysis of multivariate abundance data. *Methods in Ecology and Evolution,* 3**,** 471-474. Available from: <https://doi.org/10.1111/j.2041-210X.2012.00190.x>

Weinberger, F., Leonardi, P., Miravalles, A., Correa, J. A., Lion, U., Kloareg, B. et al. 2005. Dissection of two distinct defense-related responses to agar oligosaccharides in *Gracilaria chilensis* (Rhodophyta) and *Gracilaria conferta* (Rhodophyta). *Journal of Phycology,* 41**,** 863-873. Available from: <https://doi.org/10.1111/j.0022-3646.2005.05009.x>

Wemheuer, B. & Wemheuer, F. 2017. Assessing Bacterial and Fungal Diversity in the Plant Endosphere. *In:* STREIT, W. R. & DANIEL, R. (eds.) *Metagenomics: Methods and Protocols.* New York, NY: Springer New York. Available from: <https://doi.org/10.1007/978-1-4939-6691-2_6>

Yilmaz, P., Parfrey, L. W., Yarza, P., Gerken, J., Pruesse, E., Quast, C. et al. 2014. The SILVA and “All-species Living Tree Project (LTP)” taxonomic frameworks. *Nucleic Acids Research,* 42**,** D643-D648. Available from: <https://doi.org/10.1093/nar/gkt1209>

Zhang, B., Penton, C. R., Xue, C., Quensen, J. F., Roley, S. S., Guo, J. et al. 2017. Soil depth and crop determinants of bacterial communities under ten biofuel cropping systems. *Soil Biology and Biochemistry,* 112**,** 140-152. Available from: <https://doi.org/10.1016/j.soilbio.2017.04.019>
